# Recombination of the right cerebral cortex in patients with left side USN after stroke: fNIRS evidence from resting state

**DOI:** 10.3389/fneur.2023.1178087

**Published:** 2023-07-20

**Authors:** Shanshan Shi, Shuyan Qie, Hujun Wang, Jie Wang, Tiejun Liu

**Affiliations:** ^1^Rehabilitation Clinic, Beijing Rehabilitation Hospital, Capital Medical University, Beijing, China; ^2^Department of General Surgery, Beijing Rehabilitation Hospital, Capital Medical University, Beijing, China

**Keywords:** functional near-infrared spectroscopy (fNIRS), unilateral spatial neglect (USN), right cerebral cortex, stroke, small worldness

## Abstract

**Objective:**

Unilateral spatial neglect (USN) is an impaired contralesional stimulus detection, response, or action, causing functional disability. After a stroke, the right hemisphere experiences USN more noticeably, severely, and persistently than the left. However, few studies using fNIRS have been reported in cases of USN. This study aimed to confirm weaker RSFC in USN and investigate the potential inherent features in hemodynamic fluctuations that may be associated with USN. Furthermore, these features were combined into a mathematical model for more accurate classification.

**Methods:**

A total of 33 stroke patients with right-sided brain damage were chosen, of whom 12 had non-USN after stroke, and 21 had USN after stroke (the USN group). Graph theory was used to evaluate the hemodynamic signals of the brain's right cerebral cortex during rest. Furthermore, a support vector machine model was built to categorize the subjects into two groups based on the chosen network properties.

**Results:**

First, mean functional connectivity was lower in the USN group (0.745 ± 0.239) than in the non-USN group (0.843 ± 0.254) (*t* = −4.300, *p* < 0.001). Second, compared with the non-USN group, USN patients had a larger clustering coefficient (C) (*t* = 3.145, *p* < 0.001), local efficiency (LE) (*t* = 3.189, *p* < 0.001), and smaller global efficiency (GE) (*t* = 3.047, *p* < 0.001). Notably, there were differences in characteristic path length (L) and small worldness (σ) values between the two groups at certain thresholds, mainly as higher L (*t* = 3.074, *p* < 0.001) and lower small worldness (σ) values (*t* = 2.998, *p* < 0.001) in USN patients compared with non-USN patients. Finally, the classification accuracy of the SVM model based on AUC aC (*t* = −2.259, *p* = 0.031) and AUC aLE (*t* = −2.063, *p* = 0.048) was 85%, the sensitivity was 75%, and the specificity was 89%.

**Conclusion:**

The functional network architecture of the right cerebral cortex exhibits significant topological alterations in individuals with USN following stroke, and the sensitivity index based on the small-world property AUC may be utilized to identify these patients accurately.

## 1. Introduction

Unilateral spatial neglect (USN) is one of the most prevalent attention disorders after stroke. It mainly manifests as impairment in perception and response to contralateral stimuli, which is observed in 35% to 50% of patients after right hemispheric stroke (1–4). Notably, USN is a heterogeneous disorder characterized by different types of ignoring stimuli (1, 5): perception-attentive neglect vs. movement-intentional neglect, ignoring external personal space vs. surrounding personal space vs. personal space, or by distinct reference frames of neglect (4). Regarding frames of reference, some patients ignore the dual side of space (audience-centered or egocentric USN). In contrast, others ignore the dual side of each stimulus without regard to its position concerning the audience (stimulus-centered or distribution-centered neglect) (6–8). Persistence of USN is associated with a greater disability after stroke (1). Additionally, right-sided USN after a left-hemispheric stroke may be as common as left-sided USN after a right-hemispheric stroke (9, 10). However, right hemisphere USN was more pronounced, severe, and lasted longer than left hemisphere USN after hemilateral stroke (11, 12). According to the findings of a study involving 359 right-sided stroke patients who were undergoing rehabilitation, patients with USN (*n* = 130) had worse rehabilitation outcomes, a longer hospital stay, and more severe functional motor and cognitive impairments both at admission and discharge as determined by functionally independent measures than patients without USN (4). Thus, a thorough knowledge of the processes, predictors, and interventions that enhance USN is essential for increasing the function and quality of life after stroke.

The localization of the brain directly impacts the occurrence of USN. The severity of USN is also linked to widespread declines in resting-state functional connectivity between nodes of the attentional, motor, and auditory networks and contralateral brain regions, according to a study using resting-state functional magnetic resonance imaging (fMRI) (13). These neglect-related functional connectivity changes are mainly seen in stroke patients with right hemisphere involvement. In other words, a right hemisphere stroke is more likely to have various neural network defects on both sides, leading to USN stroke, than a left hemisphere stroke. Therefore, we focused on USN recovery following a right hemisphere stroke, congruent with much of the USN recovery and rehabilitation research.

Memory, attention, and visual processing are all parts of the frontoparietal network, also called the executive attention network. Hemineglect has been connected to the frontoparietal network, which involves both endogenous and intentional attention (14). According to electroencephalographic (EEG) testing, patients with right hemispheric damage who experienced left-sided unilateral neglect symptoms had pathologically enhanced alpha oscillations in their parietal and occipital regions (15). Furthermore, a randomized, double-blind, controlled study (16) showed that neglect brought on by right hemisphere impairment enhanced left spatial exploration. Additionally, the frequency of USN is also relatively high in cases of profound brain injury to the thalamus and basal ganglia. According to an investigation, damage to the basal ganglia and thalamus impairs the upper longitudinal tract, which in turn disrupts the connection between the parietal and frontal lobes, causing unilateral spatial neglect and impairing the function of the parietal lobe and temporal cortex (17). It should also be noted that there were few instances of unilateral spatial neglect when the lesion was limited to the subcortical white matter region (18). There are additional research findings available. Fink et al. utilized MRI to identify USN and found a direct association between subparietal lobe damage and USN, but no correlation was observed with the right parietal cortex, cerebellar vermis, or left cerebellar hemisphere (19). The etiology of unilateral neglect in the literature is unknown despite many pertinent studies. Furthermore, unilateral neglect is a heterogeneous disorder whose pathogenesis remains to be explored for better diagnosis and treatment.

Functional near-infrared spectroscopy (fNIRS) has a variety of uses in stroke research and is a valuable tool for clinical research (20, 21). fNIRS is an optical neuroimaging technique that measures changes in the concentrations of oxygenated hemoglobin (Δ[HbO_2_]) and deoxyhemoglobin (Δ[Hb]), reflecting relative regional brain activity (22). Since body motion and closed environments are not as strictly regulated as in fMRI, fNIRS can detect brain activity with high ecological validity. The benefits of fNIRS include being inexpensive, non-invasive, and portable and having high temporal and moderate spatial resolutions (22). fNIRS is also useful for resting-state (RS) and resting-state functional connectivity (RSFC) studies, as well as for detecting changes in hemodynamic signals brought on by the stimulation of neuronal activity (23, 24).

This study examined right cerebral cortex network RSFC recombination in patients with USN following stroke using fNIRS. The small-world characteristics of the right cerebral cortex brain networks in patients with USN after stroke were examined from the complex network graph theory standpoint. We employed a machine learning technique to categorize patients with and without USN following stroke based on specific network properties. Importantly, this allowed us to confirm the existence of certain right cerebral cortex network patterns in USN patients. A recent study by fNIRS on USN showed that decreased activity in the right parietal association cortex, related to spatial perception, during the prism adaptation task and task-induced reorganization of the right frontal and parietal areas were involved in the improvement of USN symptoms (25). However, few studies using fNIRS have been reported in cases of USN. Thus, the goal of this study has two aspects: in addition to confirming weaker RSFC in USN. We investigated the potential inherent features in hemodynamic fluctuations that may be associated with USN. Furthermore, we combined these features into a mathematical model to achieve a more accurate classification with higher sensitivity and specificity.

## 2. Methods

### 2.1. Subjects

This study included 33 stroke patients who received rehabilitation treatment at Beijing Rehabilitation Hospital, Capital Medical University, between 1 March 2021 and 1 July 2022, including 21 with USN (USN group) and 12 without USN (non-USN group). The inclusion criteria for subjects with stroke were as follows: (1) right unilateral stroke confirmed by MRI or CT for the first time, with an onset time of more than 14 days; (2) hemiplegia of the left limb; (3) GCS coma score ≥8; (4) sitting for 2 min or more; and (5) age: 30–80 years. The exclusion criteria were as follows: (1) unstable vital signs; (2) a metal fixator in the head and a pacemaker in the body; (3) history of seizures; and (4) visual impairment or visual defect. The inclusion criteria for the USN group were as follows: (1) USN was detected by bisection test and cancellation test of the long segment and (2) left unilateral space is ignored. The inclusion criteria of the non-USN group were as follows: (1) left hemiplegia and (2) USN is not associated with stroke. The demographic and clinical data of stroke patients are presented in Table 1. All included patients had a high school diploma of secondary education or higher. There were no significant differences in sex, age, disease course, diagnosis, or education between the two groups (*P* > 0.05). Furthermore, all subjects were informed of the experimental procedure and basic requirements before the experiment and signed an informed consent form. This study was approved by the Ethics Committee of Beijing Rehabilitation Hospital, Capital Medical University (2020bkky-034).

**Table 1:** Demographic and clinical data of stroke patients.

	**USN**	**non-USN**	**t/z**	***P***
Age (years)	56.43 ± 10.86	55.33 ± 10.99	0.278	0.783
Time post-stroke (months)	2.10 ± 0.90	2.00 ± 0.77	0.307	0.761
MMSE	22.43 ± 2.80	23.42 ± 3.12	−0.936	0.357
Sex (male/female)	16/5	9/3	0.006	0.939

USN, unilateral spatial neglect; non-USN, unilateral spatial neglect is not associated with stroke; MMSE, Mini-Mental State Examination.

### 2.2. fNIRS data collection

**Figure 1:**
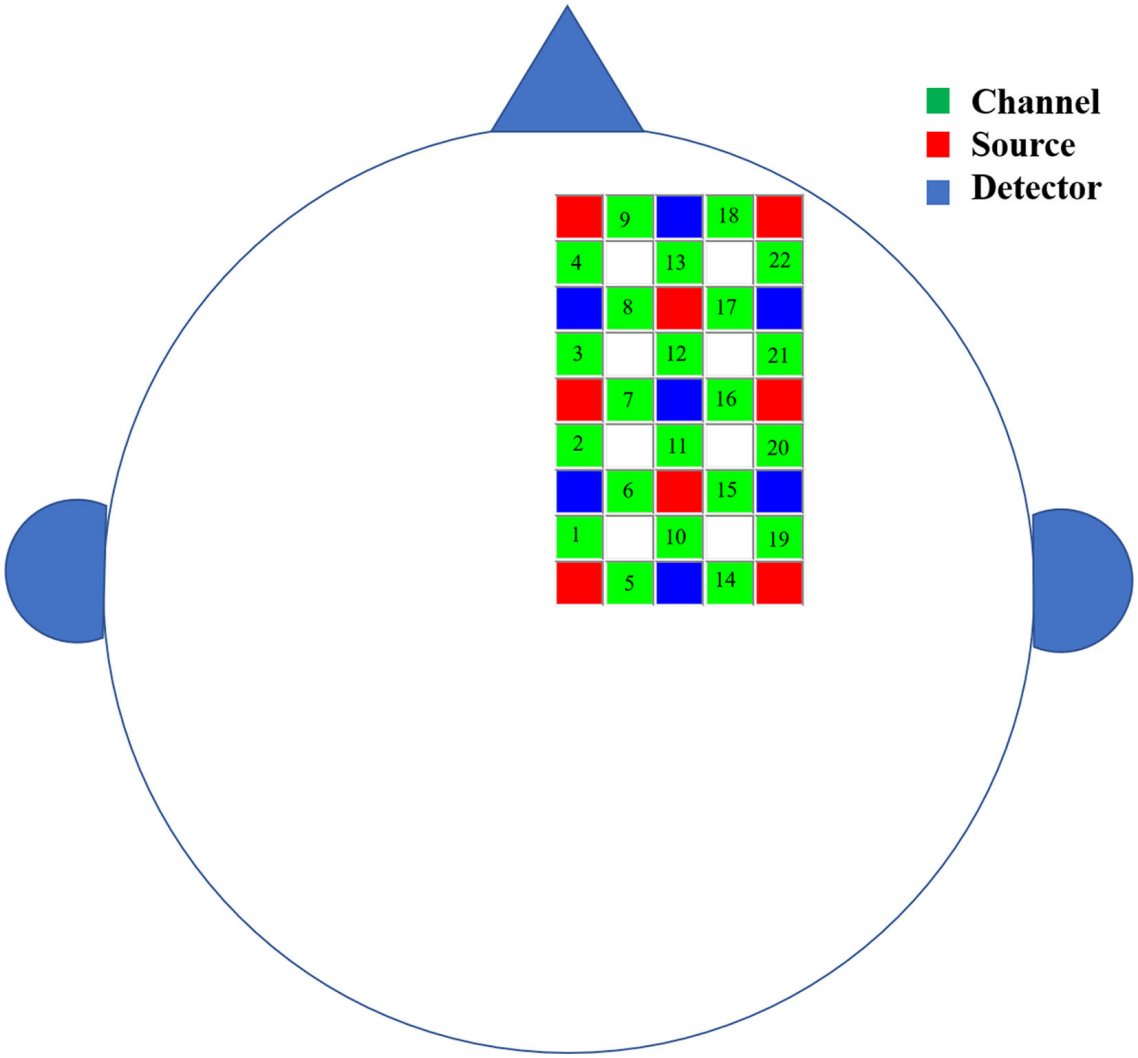
The schematic diagram of the brain area measured by fNIRS. The green squares represent channels, the red squares represent the light sources, and the blue squares represent the light detectors. In total, 8 sources and 7 detectors resulted in 22 channels covering the right cerebral region of the subject's brain.

This study used Hitachi's near-infrared functional brain imager ETG-4000 to collect the right cerebral cortex hemodynamic signals from subjects in their resting state. According to previous studies, this source-detector placement covers the right cognitive and motor-related cortex areas, including the frontal, the premotor cortex, the supplementary motor cortex, and the primary motor cortex (26–30). The positioning of the probe array was determined according to the international 10–20 coordinate system and each participant's external auditory canals and vertices were called landmarks (31). Specifically, 15 probes were attached to a soft cap designed for the subject and arranged in a 3 × 5 grid covering the right cerebral region of the subject's brain. The lower edge of the near-infrared spectral probe was flush with the subject's eyebrow bow, the upper edge was flush with the line between the two ear tips, and the medial starting point covered the Fpz position. The fNIRS probe consisted of eight light sources and seven detectors. In total, 22 detection channels were formed, and the configuration of fNIRS channels is shown in Figure 1. The distance between the light source and the detector was 3 cm. A previous study has demonstrated that near-infrared light can penetrate the cerebral cortex and can be measured by simulated scattering back to this distance (32). Furthermore, the experiment was carried out in a quiet, dark environment. In the experiment, the subjects closed their eyes, stayed awake, and avoided body movements to reduce the illusion of movement. fNIRS data were collected at a sampling rate of 10 Hz during a continuous 6-min resting period.

### 2.3. Data processing

ETG-4000 measured the attenuation changes of dual-wavelength near-infrared light (695 nm and 830 nm), and two cerebral hemodynamic parameters were obtained according to the modified Beer-Lambert law (Equationuation 1): the concentration changes of oxyhemoglobin (Δ[HbO_2_]) and deoxyhemoglobin (Δ[Hb]):


(1) $$\begin{array}{l} {\text{OD}}^{\text{λ1}}=\left(\alpha_{\text{Hb}}^{\text{λ1}}{\text{c}}_{\text{Hb}}\text{+}\alpha_{{\text{HbO}}_{\text{2}}}^{\text{λ1}}{\text{c}}_{{\text{HbO}}_{\text{2}}}\right)\times {\text{DPF}}^{\text{λ1}}\times \text{d}\\ {\text{OD}}^{\text{λ2}}=\left(\alpha_{\text{Hb}}^{\text{λ2}}{\text{c}}_{\text{Hb}}\text{+}\alpha_{{\text{HbO}}_{\text{2}}}^{\text{λ2}}{\text{c}}_{{\text{HbO}}_{\text{2}}}\right)\times {\text{DPF}}^{\text{λ2}}\times \text{d} \end{array}$$

where ΔOD is the change in light intensity attenuation, α is the specific extinction coefficient, Δc is the concentration change, DPF is the differential path-length factor, and d is the distance between the light source and the detector.

This study used the Matlab software for the offline processing of fNIRS data. First, the common average reference (CAR) spatial filtering method was used to remove the superficial interference in the hemodynamic signals (33). The CAR spatial filtering method assumes that global interference signals affect all fNIRS channels. Therefore, the superficial interference can be reduced by calculating the average of all channels and subtracting this average from every single channel and each time point. Subsequently, 0.01–0.1 Hz band-pass filtering was used to remove low-frequency drift and physiological noise (34). Following visual inspection, data segments with obvious motion artifacts were discarded. Data from each patient's first 3 s and last 3 s were also removed. Finally, for network analysis, relatively stable 3 min of hemodynamic data were selected for each patient (27, 35). Notably, only HbO_2_ results were analyzed in this study due to the higher signal-to-noise ratio for Δ[HbO_2_] than Δ[Hb] (36).

### 2.4. Small-world properties

The graph theory was used to analyze this study's small-world properties of the right cerebral cortex networks. The fNIRS channel was used as the network node, and Pearson's correlation coefficient between channels was used as the connection. By calculating Pearson's correlation coefficient between each channel pair, a 22 × 22 correlation coefficient matrix was obtained. Then, Fisher's R to Z transformation was performed on it to improve the normality. Finally, the corresponding binary network was obtained through sparseness with different thresholds, and the topological properties of the corresponding binary network under each threshold were analyzed. For this investigation, we chose a threshold range of 0.3–0.8 with a step size of 0.01 (35). All analyses were carried out over a range of thresholds because there is no “correct” threshold (37). A threshold range of 0.3 to 0.8 with a step size of 0.01 was chosen to rarefy the network; 0.3 was chosen to exclude the low-level correlation in topology, and 0.8 was chosen to reduce the data splitting (38). First, take the absolute value of the Z score matrix and then sort from the largest to the smallest. Set the connection whose absolute value is greater than a threshold value to be 1, and set the rest to be 0. For example, when a threshold value was set as 0.6, the Z score's first 40% absolute values were defined as 1, and the others were defined as 0; 1 represented the connectivity between two channels. That is, there was an edge between the two channels. This threshold-setting method can ensure that the two groups' right cerebral cortex area network properties are compared under the same connection. After constructing the sparse binary network, FC_NIRS (39) was used to calculate the clustering coefficient (C), characteristic path length (L), local efficiency (LE), global efficiency (GE), and small worldness (σ) of the right cerebral cortex area network. The node clustering coefficient (Equation 2) represents the ratio of the connection number of the neighboring node directly connected to the node to the maximum possible connection number:


(2) $$\begin{array}{l} C_{i}=\frac{2e_{i}}{n_{i}\left(n_{i}-1\right)} \end{array}$$

Among them, e_i_ represents the number of existing connections between node i and its neighbors, n_i_ represents the degree of node i, and Ci is the clustering coefficient of node i. The entire network's clustering coefficient (Equation 3) is the mean of the clustering coefficients of all nodes, and the network clustering coefficient represents the degree of local clustering of the network:


(3) $$\begin{array}{l} C=\frac{1}{N}\overset{N}{\underset{i=1}{\sum }}C_{i} \end{array}$$

The characteristic path length (Equation 4) is the mean value of the shortest path of all possible pairs of nodes in the network. The characteristic path length also reflects the information transmission efficiency of the network and the degree of network integration:


(4) $$\begin{array}{l} L=\frac{1}{N\left(N-1\right)}\underset{i,j\in N,i\neq j}{\sum }d_{i,j} \end{array}$$

Among them, d_i, j_ is the shortest path length between nodes i and j. The global effect and the local effect are measures of network efficiency. The global effect (Equation 5) is the mean value of the reciprocal of the shortest path length (d_i, j_) of all nodes:


(5) $$\begin{array}{l} E_{glob}=\frac{1}{N\left(N-1\right)}\underset{i,j\in N,i\neq j}{\sum }\frac{1}{d_{i,j}} \end{array}$$

Local efficiency (Equation 6) is the average of the efficiency of the subgraph Gi composed of all neighbor nodes of each node in the network:


(6) $$\begin{array}{l} E_{loca}=\frac{1}{N\left(N-1\right)}\underset{i=1}{\sum }E\left(G_{i}\right) \end{array}$$

In network analysis, we first obtained the small worldness σ (Equation 9) according to the normalized C (γ, Equation 7) and the normalized L (λ, Equation 8). We checked whether the right cerebral cortex area network had small-world characteristics. Subsequently, we analyzed the differences in five small-world attributes (C, L, LE, GE, and σ) between the two groups of subjects:


(7) $$\begin{array}{l} \gamma =C_{\text{real}}/C_{\text{rand}} \end{array}$$

(8) $$\begin{array}{l} \lambda =L_{\text{real}}/L_{\text{rand}} \end{array}$$

(9) $$\begin{array}{l} \delta =\gamma /\lambda \end{array}$$

Among them, C_real_ and L_real_ are the C and L of the real network, respectively, and Crand and L_rand_ are the average C and the average L of 100 random networks.

### 2.5. The right cerebral cortex areas network pattern classification

To explore whether the patient has a specific right cerebral cortex area network pattern that is different from normal subjects, we selected the right cerebral cortex area network features. We then used the machine learning method to classify the two groups of subjects. The essence of pattern recognition is to classify the input pattern into a predefined category or realize the automatic classification of the pattern according to the similarity between patterns (40). If a stroke patient has a specific brain network pattern different from a healthy individual, machine learning can reasonably distinguish the two. To avoid the influence of the threshold, we used the area under the curve (AUC) of the five network parameters under the threshold of 0.3–0.8 as alternative features. AUC can provide a summarized scalar for the topological characteristics and is independent of selecting a single threshold (41, 42). In addition, AUC is sensitive to changes in brain network topology (43). The AUCs of the five network parameters were compared between two groups with *t*-tests, and then AUCs that were significantly different between the two groups (P < 0.05) were selected as classification features. Based on the selected features, a support vector machine (SVM) model was established to classify the two groups of subjects. SVM is derived based on the principle of structured risk minimization. The optimal decision plane of SVM depends on the support vector and does not depend on the sample size, so it is suitable for small sample classification. Moreover, grid search was used to optimize the SVM model's penalty coefficient and kernel function parameters. The optimal model parameters in this study were C = 3 and kernel = “linear.” We used leave-one-out cross-validation to evaluate the model performance, leaving one subject's data for model testing and the other for model training. Furthermore, this step was repeated until each subject had been tested once. Finally, the performance of the model was evaluated through the test results for each subject by sensitivity (Equation 10), specificity (Equation 11), and accuracy (Equation 12):


(10) $$\begin{array}{l} Sensitivity=TP/\left(TP+FN\right) \end{array}$$

(11) $$\begin{array}{l} Specificity=TN/\left(FP+TN\right) \end{array}$$

(12) $$\begin{array}{l} Accuracy=\left(TP+TN\right)/\left(TP+TN+FP+FN\right) \end{array}$$

Among them, false negative (FN) is the number of misclassified USN patients. False positives (FP) are the number of misclassified non-USN subjects. True positive (TP) is the number of USN patients correctly classified, and true negative (TN) is the number of non-USN subjects correctly classified.

### 2.6. Statistical analysis

The MATLAB software was used for Pearson's correlation analysis and *t*-tests. Additionally, all *t*-tests were corrected by FDR. The comparison of FC between the two groups was conducted, the correlation matrices of all participants in a family were averaged, and the average connectivity between the two groups was compared using the *t*-test. The comparison of small-world attributes between the two groups was conducted using the two-independent sample *t*-test. Statistical analysis was performed using SPSS 22.0. The Python software was used for SVM modeling and classification prediction. The statistical significance level was set at 0.05.

## 3. Results

### 3.1. Functional connectivity

**Figure 2:**
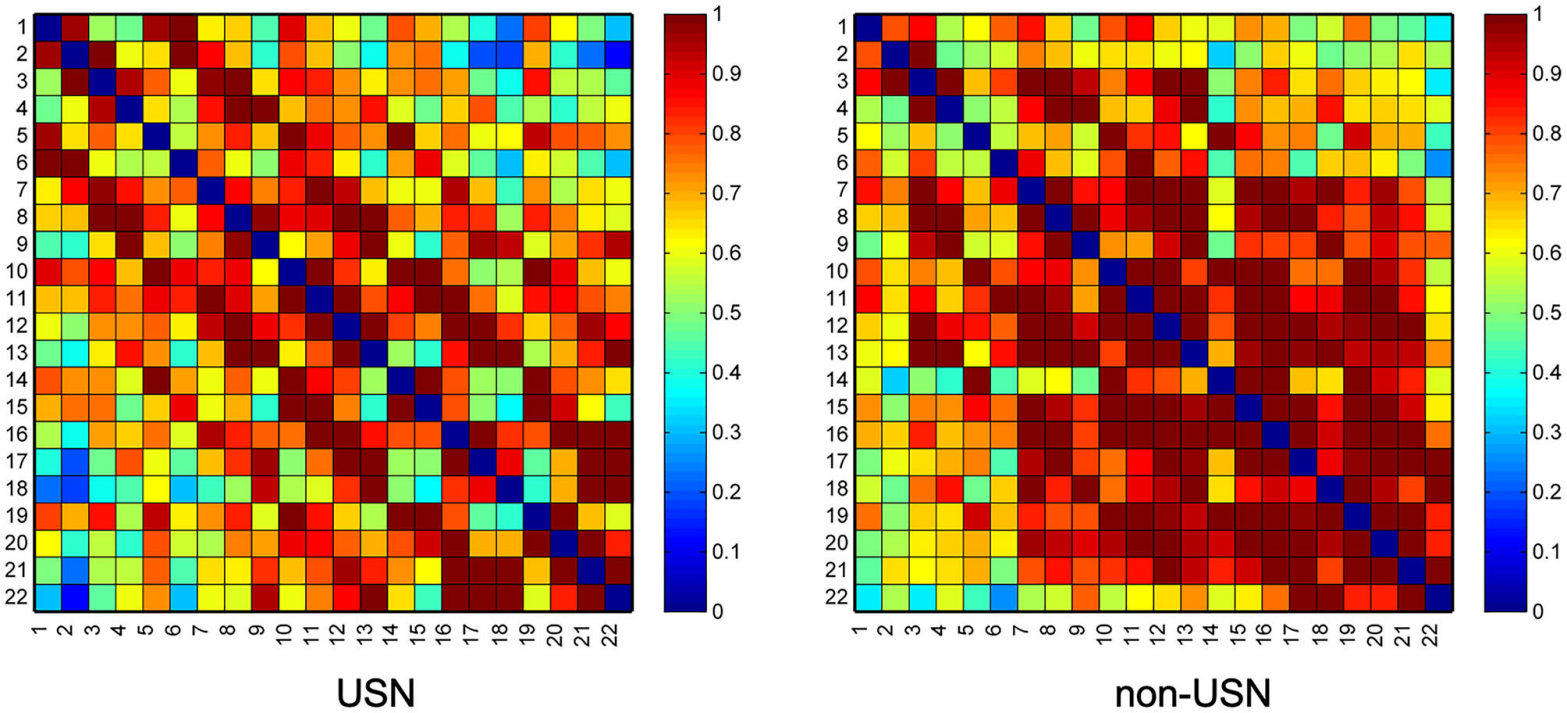
Two groups' resting-state functional connectivity (RSFC) matrix diagram. Axes represent the channels—each channel with its correlation coefficient set at zero (the diagonal line).

The average group FC of all groups is shown in Figure 2. There was a statistically significant difference in FC values between the two groups (*t* = −4.300, *p* < 0.001), and the mean functional connectivity intensity in the USN group (0.745 ± 0.239) was lower than that in the non-USN group (0.843 ± 0.254).

### 3.2. The right cerebral cortex network features

**Figure 3:**
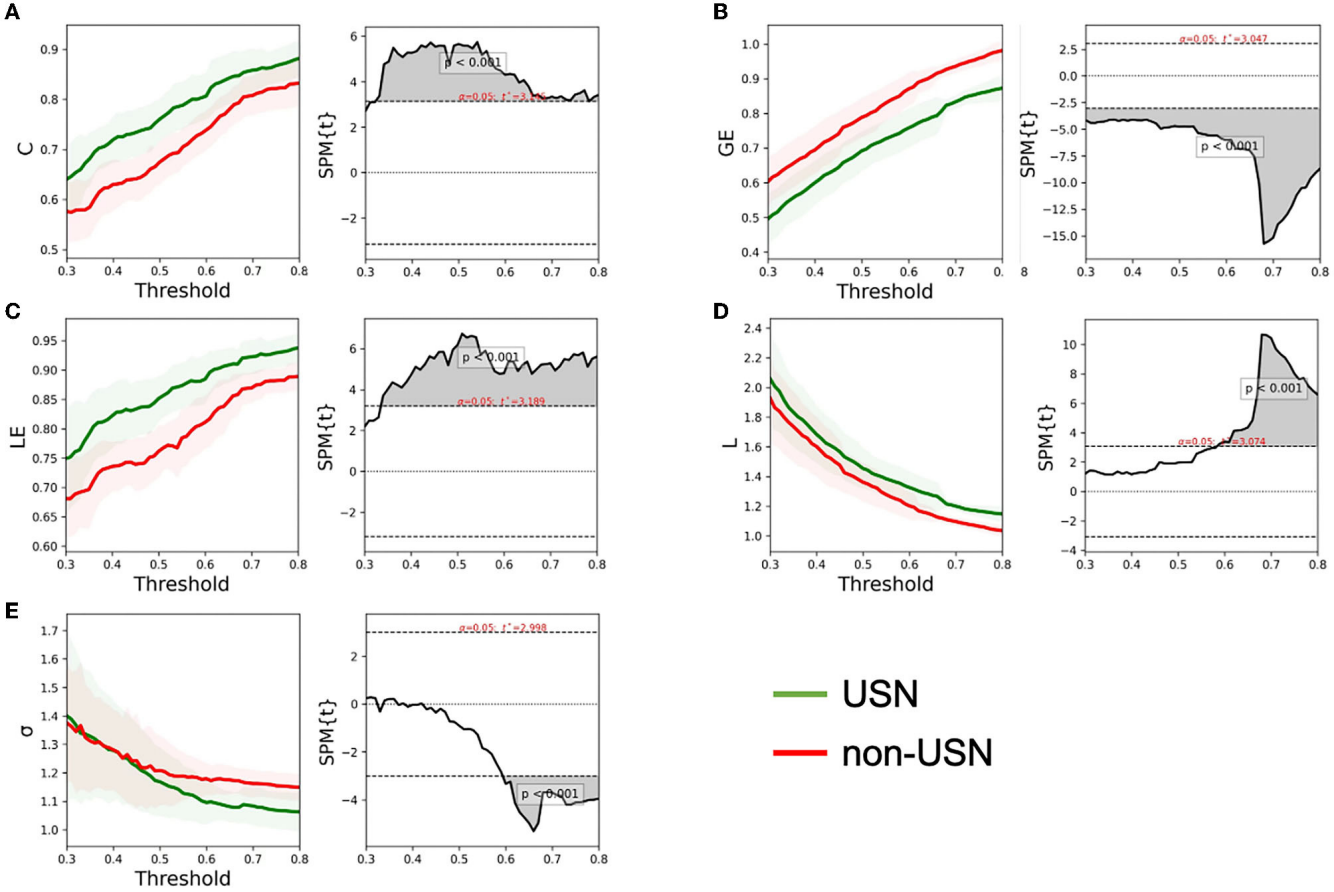
The global network metrics in a range of sparsity thresholds (30–80%). The mean C **(A)**, GE **(B)**, LE **(C)**, L **(D)**, and σ **(E)** of the two groups of subjects under each threshold. The shadow on the left of each figure represents the standard error for all participants, and the gray shadow on the right represents the threshold range with significant differences between the two groups.

Figure 3 shows the changing trend of the small-world attribute of the right cerebral cortex network for the two groups of subjects under the threshold of 0.3–0.8. With the increase of threshold, clustering coefficient **(C)**, local efficiency (LE), and global efficiency (GE) all increased. The characteristic path length **(L)** and small worldness (σ) value decreased with the increase in the threshold. The first objective of this study was to investigate right cerebral cortex network recombination in patients with USN after stroke. To this end, we studied the difference between the two groups of subjects based on the topological properties of the right cerebral cortex networks. If the network has small-world properties, small worldness (σ) should be larger than 1 (44). Figure 3 shows that both groups' right cerebral cortex networks showed small-world characteristics. *T*-test results showed that C (*t* = 3.145, *p* < 0.001), LE (*t* = 3.189, *p* < 0.001), and L (*t* = 3.074, *p* < 0.001) in patients with USN after stroke were higher than those with stroke. Additionally, GE (*t* = 3.047, *p* < 0.001) in patients with USN was lower than in those with stroke. Furthermore, L and small worldness σ values were different between the two groups under certain thresholds, mainly showing that L in USN patients was higher than that in non-USN patients (*t* = 3.074, *p* < 0.001), and small worldness σ value in USN patients was lower than that in non-USN patients (*t* = 2.998, *p* < 0.001) (Figure 3).

### 3.3. SVM classification results

**Figure 4:**
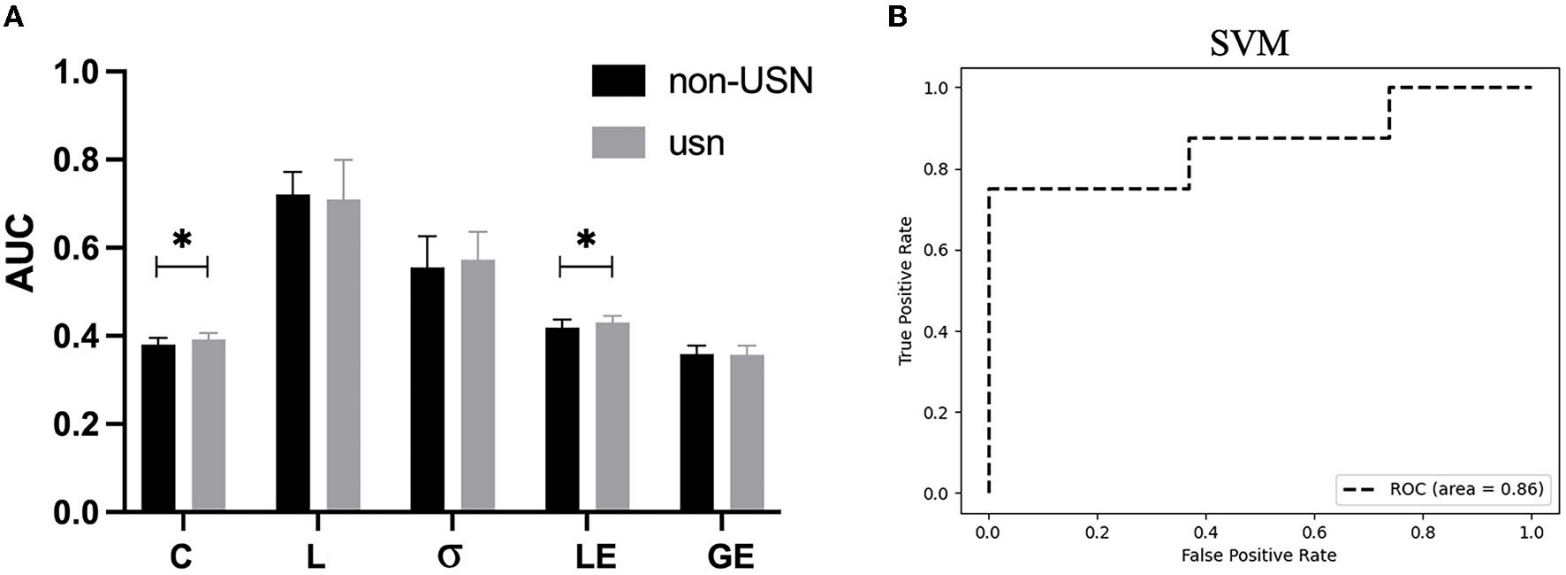
**(A)** The AUC indicators for the five small-world attributes of the two groups of subjects, in which AUCs for C, L, δ, LE, and GE are significantly different between the two groups. **(B)** The ROC curve of the SVM model for the two groups. *p < 0.05.

According to the *t*-test results of the five small-world attributes between the two groups, we chose the clustering coefficient AUC (*t* = −2.259, *p* =0.031) and global effect AUC (*t* = −2.063, *p* = 0.048)as the classification features (Figure 4A). The established SVM model had a classification accuracy rate of 85% for the two groups of subjects. The sensitivity and specificity were also 75% and 89%, respectively. Furthermore, the SVM model's receiver operator characteristic (ROC) curve for classifying two groups is shown in Figure 4B, with the AUC reaching 0.85.

## 4. Discussion

In the last two decades, the interest in fNIRS has been gradually evolving for its real-time monitoring, relatively low-cost, radiation-less environment, portability, patient-friendliness, etc. Including brain-computer interface and functional neuroimaging research, this technique has some important applications of clinical perspectives, such as Alzheimer's disease, schizophrenia, dyslexia, Parkinson's disease, childhood disorders, post-neurosurgery dysfunction, attention, and functional connectivity can be diagnosed in some form of assistive modality in clinical approaches (45). However, studies using near-infrared spectroscopy in USN cases are rarely reported. At present, the pathogenesis of USN is unknown, and diagnosis only depends on the scale, while those with cognitive dysfunction or language impairment of USN patients are not timely diagnosed. In this study, we employed a graph theory approach to investigate the reorganization of the brain network in patients with USN and aimed to establish a machine learning-based assessment model for predicting USN. In this study, resting fNIRS was tested in the right cerebral cortex in patients with and without USN after stroke, and the key findings include (1) functional network connectivity in the right cerebral cortex was lower in patients with unilateral neglect than in the non-USN group; (2) the functional brain networks of the stroke patients showed uniformly comparable small-world economic organization; (3) compared with stroke patients without unilateral neglect, USN patients showed lower global efficiency and a smaller world; USN patients also showed higher clustering coefficient, higher local efficiency, and higher feature path length; and (4) based on the selected network features, the established SVM model was used to classify the two groups of subjects with 85% accuracy. Overall, we observed significant changes in the topological organization of the right cerebral cortex network in patients with unilateral neglect after stroke. Notably, our results provide insights into the critical topological changes in functional brain networks in patients with USN. These results are further discussed in detail below.

RS is an organized baseline state or default mode of brain function, and resting-state functional connectivity (RSFC) can provide meaningful information about cortical restructuring after stroke. RSFC is closely related to poststroke functional status (46) and has the potential to predict poststroke recovery (47). Importantly, RSFC is a promising poststroke brain functional status biomarker and is primarily used in clinical trials (48). Previous studies have shown a general reorganization of brain function at RS after stroke (49). In this study, fNIRS tests were performed on the right cerebral cortex network of patients from the stroke group and the USN group, suggesting that the right cerebral cortex network in the USN group not only reorganized but also showed weaker functional connectivity than in the stroke group. Furthermore, the right cerebral cortex network pattern was specific in patients with USN after stroke compared with patients without USN.

Functional recombination of the prefrontal cortex after stroke is associated with several functions in patients. PFC is associated with cognitive function (13) and emotional recovery (50) in stroke patients. PFC is also important for motor control in stroke patients, allowing them to prioritize walking (51). In some studies, damage to the basal ganglia and thalamus has been found to affect the upper longitudinal tract, disrupting the connection between the parietal and frontal area and resulting in unilateral spatial neglect (24).

This study aimed to investigate the recombination of the right cerebral cortex network at RS in patients with USN after stroke and to analyze the topological nature of the right cerebral cortex network. The results showed that the right cerebral cortex network has small-world properties in patients with and without USN after stroke. Furthermore, brain networks with small-world properties may provide a topological basis for local specialization and globally distributed processing (52). In this study, the topological properties of the right cerebral cortex network were significantly different between USN patients after stroke and those without USN at RS. The clustering coefficient of the right cerebral cortex network in USN patients after stroke increased, indicating that the number of local short connections in the right cerebral cortex network increased in USN patients, and strong local clustering occurred. After a stroke, the whole brain network was reorganized due to neuronal disruption (53, 54). Although most stroke patients in this study had brain lesions not located in the right cerebral cortex, the right cerebral cortex network was reorganized during USN recovery after stroke, and this recombination pattern was very specific. In this study, the clustering coefficient, local efficiency, and characteristic path length of the right cerebral cortex network were higher in patients with USN after stroke than in patients without USN. In contrast, the global efficiency and small-world attribute were lower than in patients without USN. The reduction of long-term connections in the right cerebral cortex network in USN patients after stroke (the LE_AUC was higher in USN patients than in the stroke group) suggests that the isolation between the local components of the stroke degree is higher in stroke patients, which reduces the overall efficiency and small world of the PFC network (55). Local efficiency was higher in stroke patients than in healthy controls, and global efficiency was lower than in healthy controls (56). Our findings are in line with Li et al. study, which demonstrated that the brain networks of acute stroke patients exhibit reduced cosmopolitan structure and fewer long-range connections compared to those of healthy individuals (57). Similar research has discovered a correlation between small-world characteristics in the inferior cerebral cortex region of RS and motor function among patients (58). Studies have demonstrated that there is an inverse relationship between motor function and the clustering coefficient of the brain network (35). This study revealed a larger clustering coefficient in the right cerebral cortex network of USN patients compared to the non-USN group, indicating poor exercise ability consistent with previous research findings (1, 59–61).

Machine learning has already been widely applied in the field of medicine due to its capacity for identifying discriminant variables that can be utilized for making predictions (62), as well as its ability to easily integrate new data and enhance predictive performance (63). In stroke research, machine learning has improved the assessment and prediction of diagnostic and therapeutic purposes (64, 65). The objective of this study was to develop a machine learning-based assessment method that provides an objective evaluation of brain network reorganization. Based on the characteristics of the selected brain network, the SVM model exhibited high sensitivity and specificity for both subject groups, with a higher sensitivity than specificity. This may be attributed to the larger sample size of patients in the USN group compared to that of the non-USN group and lateralization during classification. Additionally, it is possible that there exists a similar network pattern within the right cerebral cortex brains among USN patients after stroke, which reduces variability.

This study also has noted limitations. First, the number of subjects in this study was relatively small, with only 12 non-USN subjects and 21 USN subjects. In the future, we will recruit more subjects to further investigate the clinical value of right cerebral cortex network features in patients with USN after stroke. Additionally, because this was a preliminary study, only the right side of the patients' brains was examined, and the test site was limited. Given that the unilaterally disregarded injury site is not only confined to the right hemisphere or cerebral cortex but also encompasses regions such as the parietal lobe, occipital lobe, and basal ganglia, future investigations should employ more comprehensive and rigorous testing to further elucidate the pathogenesis of USN.

## 5. Conclusion

This study represents the first attempt to investigate the level of brain function in the right cerebral cortex region among patients with unilateral spatial neglect using fNIRS analysis of resting-state brain networks. Our findings demonstrate significant topological alterations in the functional network organization of the right cerebral cortex among stroke patients with unilateral spatial neglect, compared to those without such neglect. These results offer novel insights into subtle changes in the pathogenesis of USN brain functional networks.

## Data availability statement

The raw data supporting the conclusions of this article will be made available by the authors, without undue reservation.

## Ethics statement

The studies involving human participants were reviewed and approved by the Ethics Committee of Beijing Rehabilitation Hospital, Capital Medical University (2020bkky-034). The patients/participants provided their written informed consent to participate in this study.

## Author contributions

SS and SQ were responsible for the experimental design. SS, HW, and JW took charge of the data analysis. TL made the final decisions during the whole process. All authors participated in the implementation of the experiment and the writing of this article and contributed to the article and approved the submitted version.
